# Generating synthetic journal entries for audit analytics: Method and dataset

**DOI:** 10.1016/j.dib.2026.113257

**Published:** 2026-09-11

**Authors:** Jan Gronewald, Sebastian Stephan, Alexander Rombach, Peter Fettke

**Affiliations:** a German Research Center for Artificial Intelligence (DFKI), Campus D3.2, 66123, Saarbruecken, Germany; b Saarland University, Campus D3.2, 66123, Saarbruecken, Germany

**Keywords:** Financial auditing, General ledger, Journal entry testing, Fraud detection, Anomaly detection, Synthetic data

## Abstract

This article presents a labeled synthetic general ledger dataset created for journal entry testing in audit analytics. Journal entry testing is a use case of financial audit that deals with the detection of erroneous or fraudulent entries in the general ledger. The dataset represents two fiscal years of transactions for a fictional company as recorded in an accounting system. The postings are generated to comply with the rules of double-entry bookkeeping, with each journal entry consisting of at least two posting lines. In addition to regular transactions, the dataset contains transactions that would be classified as problematic in the annual audit. The synthetic postings were generated using predefined transaction patterns and probabilistic distributions for transaction frequency and other attribute values. The dataset can be reused for the development and benchmarking of supervised, unsupervised, rule-based, and hybrid approaches to anomaly detection in accounting data. The generation workflow can also serve as a reusable blueprint for constructing synthetic accounting datasets in related contexts.

Specifications Table

**Table:** 

Subject	Computer Sciences
Specific subject area	Synthetically generated general ledger data with curated anomalies.
Type of data	CSV file of raw synthetic accounting data (general ledger).
Data collection	The dataset was synthetically generated and is based on a fictional company and its general ledger. To this end, typical business transactions were defined to simulate two fiscal years of transactions and the corresponding general ledger information.
Data source location	Institution: German Research Center for Artificial Intelligence (DFKI). City/Town/Region: Saarbruecken. Country: Germany.
Data accessibility	Repository name: DIB General Ledger Data Data identification number: DOI: 10.5281/zenodo.22,014,653 Direct URL to data: https://doi.org/10.5281/zenodo.22014653
Related research article	none

## 1 Value of the Data

• The dataset represents a company's transactions during two fiscal years, as recorded in the accounting system. Companies are legally required to record these transactions, which are intended to systematically document and evaluate financial transactions. They form the basis for preparing annual financial statements and tax returns. Internal, tax and financial audits refer to this data to detect errors and fraud. As this data is sensitive for companies, there is little to no interest in publishing real accounting data. With this dataset, we publish synthetic data that illustrates the nature of general ledger data and is usable for research and practice.
• In addition to the synthesized business transactions, the dataset contains various “problematic” business transactions that should be detected during an audit.
• Our dataset is a resource for research and practice that can be used to develop, test, and compare novel methods that help improve both effectiveness and efficiency in audit analytics.
• The dataset can be used to apply anomaly detection methods to a field where no public datasets exist and where collecting real data is difficult, even in industrial projects. By providing a fully reproducible data generation process, the dataset also enables replicable and comparable research.
• In addition, our approach can be used as a general blueprint for generating artificial general ledger data records.

## 2 Background

The dataset was compiled in the context of our research on financial audit. It is designed to meet the requirements of a company’s general ledger and the use case of journal entry testing (JET) as required by the International Standards on Auditing (ISA 240) [1]. The use case covers the examination of general ledger data for risks of material misstatement due to fraud or error. According to a survey by the ACFE, organizations lose around 5% of their annual revenue to fraud [2]. In extreme cases, such as Wirecard or Enron, this can lead to insolvency. Typically, JETs are conducted using rule-based procedures derived from auditing practices and known risk patterns. In current research, various machine learning methods are discussed as a means of supplementing or improving these procedures to detect fraud cases as early as possible [[3], [4], [5], [6], [7], [8]]. To our knowledge, the tests and comparisons carried out are based solely on non-public datasets and therefore cannot be reproduced. Furthermore, obtaining such datasets for research purposes is difficult. Against this background, our dataset [9] was generated to support the systematic evaluation of machine learning approaches under controlled conditions.

## 3 Data Description

The dataset consists of synthetically generated general ledger journal entry postings that replicate realistic accounting transaction structures in a double-entry bookkeeping environment. The primary dataset is provided in the file *journal_entries_4203.csv*, which contains all simulated journal entry line items. Each row represents a single account posting, while complete journal entries are formed by grouping postings via a shared *posting_id*. Debit and credit positions are explicitly distinguished, ensuring that each grouped transaction is balanced. The final dataset consists of 19 data fields. Three of these fields are of the data type “Date”, ten are of the data type “Number”, three are of the data type “Alphanumeric” and three are of the data type “Text”. The individual fields are structured as follows: The attribute *posting_id* (alphanumeric) uniquely identifies, and groups related journal entry lines belonging to the same journal entry. The attributes *document_date* and *posting_date* (date) are manually set by the user and represent the original document date (e.g., invoice date) and the accounting period assignment date respectively. The *entered_date* (date) is system-generated and records the actual timestamp at which the posting was entered. Financial account information is captured through *gl_account* (number) and *gl_account_name* (alphanumeric), specifying the affected general ledger account. The *amount* (number) represents the net posting value, while *cd_flag* (text) indicates whether the *amount* is recorded as a debit (“D”) or credit (“C”). The *tax_rate* (number) captures the applied tax percentage. The attribute *ref_id* (alphanumeric) links postings to related clearing transactions, such as payments offsetting open invoices. The *text* field (text) contains the user-provided posting description. The *user* attribute (text) identifies the individual entering the posting.

Several attributes provide temporal and behavioral context that is derived from the entry timestamps rather than posted by the user and is supplied here in pre-computed form for analytical purposes. The attribute *promptly* (number) classifies postings according to entry timeliness (i.e., prompt, delayed, or very delayed), *weekend* (number) distinguishes entries made on weekdays, Saturdays, and Sundays, and the binary attribute *nwh* (number, “non-working hours”) identifies postings entered outside regular business hours. These attributes allow the analysis of irregular booking patterns and user behavior. To facilitate audit analytics and anomaly detection research, artificial irregularities are embedded and explicitly labeled: the attribute *label* (number) marks whether a posting belongs to an artificially inserted anomaly, while *marking* (number) links affected postings to specific anomaly scenarios along the applied JET. Additionally, the dataset includes audit-scope indicators: *top_n* (number) identifies postings falling within predefined high-value selection criteria, and *high_cash* (number) flags cash postings above a materiality threshold. Table 1 below provides an overview of the data fields.

**Table 1:** Data fields of the synthetic general ledger dataset journal_entries_4203.csv. For each of the 19 attributes, the table reports the field name, its meaning in the accounting context, and its data type.

Name	Meaning in the accounting context	Datatype
posting_id	Unique ID to group account postings together.	alphanumeric
document_date	Manually set by the user. Corresponds to the date of the document to be posted (e.g., invoice date).	date
posting_date	Manually set by the user and is used to assign the account posting to the posting period (service date).	date
entered_date	Actual date and time when the document was entered by a user. Assigned by the system.	date
gl_account	Account number.	number
gl_account_name	Name of the account used.	alphanumeric
amount	Net amount.	number
cd_flag	Credit-/debit-flag. Used to determine on which side the “gl_account” is posted (“C” / “D”).	text
user	Name of the user.	text
tax_rate	Applied tax rate on the amount.	number
ref_id	Used to refer to the posting which it clears.	alphanumeric
text	Posting text.	text
promptly	Labels account posting if it was entered promptly, delayed, or very delayed.	number
weekend	Labels an account posting if it was posted on a weekend.	number
nwh	“Non-working hours” labels an account posting if it is outside the regular working hours.	number
label	Used to mark artificially inserted anomalies.	number
marking	ID of the anomaly type.	number
top_n	Audit scope indicator describing which entries fall within the JET relating to high amounts (here: the 1000 highest postings per fiscal year).	number
high_cash	Audit scope indicator describing which entries fall within the JET detecting high cash disbursements (here: cash postings above EUR 1500).	number

The dataset is designed to preserve core accounting principles. All postings grouped under the same *posting_id* are balanced at the journal entry level, satisfying the double-entry constraint. Multiple users, tax treatments, diverse account types, and realistic temporal distributions are incorporated to approximate operational accounting environments. The dataset covers period-end effects through one accrual and one deferral, and correction patterns through reversals and credit notes. Both remain deliberately small in scale: they are instantiated at a single fiscal-year boundary, the follow-up entries are derived from purchase invoices of the closing year only (here: December), and the recurring expense block is generated outside the cross-period injection. Explicit anomaly labeling enables supervised and unsupervised learning applications.

## 4 Experimental Design, Materials and Methods

The synthetic data generation process is implemented in *synthetic_data_4203.ipynb*. This Jupyter notebook contains the full code used to simulate transactional behavior, assign contextual and temporal attributes, generate audit-relevant indicators, and inject artificial anomalies. In addition to purchase, sales, and payment transactions, the generated data comprises various expense postings as well as period-end adjustments (accrual, deferral) and corrections of existing invoices (reversal, credit note), which increase structural realism and variability. An overview of the file inventory is provided in Table 2.

**Table 2:** Inventory of the files provided in the data repository. The dataset is available at https://doi.org/10.5281/zenodo.22014653.

Path	File type	Role	Content summary
journal_entries_4203.csv	CSV	Dataset	Synthetic GL journal entry postings (line items). One row equals one posting line item; journal entries are formed by grouping rows by *posting_id*.
synthetic_data_4203.ipynb	Jupyter Notebook	Generation workflow	Code that generates synthetic postings, assigns contextual and temporal attributes, and writes journal_entries_4203.csv.

In the following, we provide general information regarding the posting context and the accounting events used to generate synthetic general ledger data (journal entries). We then describe our synthetic data generation pipeline, the standard business cases we have considered, and how not only postings, but also synthetic anomalies (anomalous postings) were generated.

### 4.1 Business case

The case study describes a fictional trading company for gardening tools. For the sake of simplicity, we focused only on the following six business cases: (1) purchase of merchandise, (2) sale of goods, (3) payments (incoming and outgoing), (4) various expenses, (5) period-end adjustments, and (6) corrections of already recorded invoices. In (4), postings such as monthly recurring expenses from payroll, expenses from various leasing agreements, service contracts, rents and other expenses for the operation of the business premises are included. In (5), an accrual and a deferral are recorded at the fiscal-year boundary, each including its settlement in the subsequent year: the accrual covers estimated December utilities and is settled by the actual invoice, whose difference to the estimate is expensed as a prior-period item; the deferral covers the share of a maintenance contract that belongs to the new fiscal year and is released at its beginning. In (6), reversals and credit notes are derived from purchase invoices of the closing year and reference the original entry via *ref_id*: a reversal cancels the original in full, in which case the payment of the original is dropped, whereas a credit note reduces it by a share of its amount and the remaining amount is paid. Both (5) and (6) are regular business transactions and they are excluded from the anomaly injection described below and carry label = 0. In total, there are six accountants who are responsible for the posting of the described business cases; one of them, Fred, also serves as the unusual user to whom the anomaly injection reassigns entries. Which accountant posts a given transaction, and how often, is determined randomly. We used a generic chart of accounts. The simulation covers two consecutive fiscal years. To reflect the accounting complexity arising at the fiscal-year boundary, a share of the year-end transactions is assigned to the closing period but entered in the following one, with the corresponding payments cleared accordingly in the new fiscal year.

To embed audit-relevant characteristics into the dataset, four JETs were defined in alignment with the simulated business processes. Their specification follows [10] and incorporates insights from expert interviews. The implemented JETs comprise: (J1) top-n highest postings, (J2) high cash disbursements, (J3) delayed postings, and (J4) postings outside regular working hours or on weekends. Assuming that postings are only made during regular working hours and that only the six accountants are allowed to post business transactions, we have simulated regular postings, which can, but do not have to, fall within the range of our JET patterns (J1-J4). In addition, two anomaly patterns were embedded that lie outside conventional JET selections: (A1) postings routed through a rarely used second bank account, and (A2) clearing postings whose reference links to a business transaction of an unrelated category.

### 4.2 Generation of synthetic data and anomalies

The literature discusses many approaches to synthetic data generation [11]. Our approach is based on the general accounting logic. The definition and simulation of postings is based on domain expertise and standard accounting principles. We focused on the generation of dedicated business transactions following the double-entry bookkeeping posting structure. Thus, a multi-line representation was selected to define individual posting records. Related posting records are identified by their *posting_id.* For generating account postings, we made different assumptions. First, we generated journal entries for two consecutive fiscal years. Second, to differentiate between items that are purchased, paid, and resold, we defined a static price list for each item. The purchase price was derived from the material costs of each item. The selling price was calculated based on the purchase price by multiplying it by a static factor; in the second fiscal year, both prices were scaled by a drift factor of 1.03 to reflect inflation. Third, timestamps, time intervals, quantities, and user assignments were drawn from probability distributions. Table 3 specifies these distributions, whereas Table 4 maps the generated variables or processes to each simulation case. The case-specific parameter values are listed in Table 6.

**Table 3:** Formal specification of the generation distributions.

ID	Generated variable or process	Formal specification	Scope
T1	Candidate timestamp pool and weighted entry time	For group g and calendar day d: N_g,d_ ∼ Poisson(15) on Monday-Friday; N_g,d_ = B_g,d_ max(0, floor(Z_g,d_)) on Saturday/Sunday, with B_g,d_ ∼ Bernoulli(ρ_g_) and Z_g,d_ ∼ N(10, 2^2^). H ∼ DU{8,…,16} with probability 1-ν_g_; DU{5,…,7} with probability ν_g_/2; DU{17,…,23} with probability ν_g_/2. Minutes and seconds are independent DU{0,…,59}. Conditional on the realized pool T_g_: Pr(E_i_=τ | T_g_) = w_wd,g_(τ) w_mo,g_(τ) / Σ_τ′∈Tg_ w_wd,g_(τ′) w_mo,g_(τ′). Sampling is with replacement.	Original entries in Cases 1.1–1.18
T2	Document/posting lag and calendar correction	X_i_ ∼ TN[−10,0](−5, 2^2^) with probability 1-λ_g_, and X_i_ ∼ U(−60,−10) with probability λ_g_; Δ_i_ = trunc₀(X_i_) whole days. P_i_ = D_i_ = R_y_(date(E_i_) + Δ_i_). R_y_ replaces a Saturday/Sunday by a uniformly selected Monday-Friday of the same week; a date outside fiscal year y is replaced by date(E_i_).	Original entries in Cases 1.1–1.18
T3	Linked payment/clearing entry	Let Δ_i1_ and Δ_i2_ be independent T2 normal-component draws (λ=0). Then E_i_^clr^ = E_i_^orig^ - Δ_i1_ and P_i_^clr^ = D_i_^clr^ = R_y_(date(E_i_^clr^) + Δ_i2_). User, tax rate, text, and item context are inherited from the original; the two clearing lines equal the original gross balance. Since Δ_i1_ is a whole-day offset, E_i_^clr^ inherits the time of day of E_i_^orig^, including non-working-hour times generated under T1.	Cases 1.2, 1.4, …, 1.18
T4	Item, quantity, user, and amount	K_i_ ∼ DU(K), |K|=10; U_i_ ∼ DU(U_g_). Q_i_=1 for a single-item case, Q_i_ ∼ DU{1,…,20} for multiple purchases, and Q_i_ ∼ DU{1,…,5} for multiple sales. A_i_^net^ = round₂(Q_i_ π_i_ δ_y_), where π_i_ is the configured purchase cost or sales price of K_i_ for the applicable case, δ_2023_=1, and δ_2024_=1.03. Tax, gross amount, and balanced debit/credit lines are deterministic functions of A_i_^net^ and the tax rate.	Purchase and sales originals
T5	Cross-period selection and transformation	S_g_ ∼ SRSWOR(C_g_, n_g_). For i∈S_g_, the original posting/document dates and non-temporal attributes are retained; E_i_ is replaced by a timestamp with an equiprobable day in [a_g_,b_g_], H ∼ DU{8,…,16}, and minute/second ∼ DU{0,…,59}. Each linked clearing entry is moved to E_i_ + L_i_, with L_i_ ∼ DU{ℓ_g_^min^,…,ℓ_g_^max^}; its time of day is redrawn as above and its posting/document date is set to the resulting calendar date. No weekend correction is applied in this transformation. The three cases are applied sequentially to the same journal in the order 1.19, 1.20, 1.21, and each candidate set excludes originals whose entry date an earlier case has already moved.	Cases 1.19–1.21
T6	Scheduled payroll and recurring expenses	Payroll follows a deterministic monthly schedule and fixed employee-specific amounts; dates are advanced to the next Monday-Friday when necessary. For recurring expense type j in scheduled month m: d_jm_* = bd_y_(d_jm_^0^ + J_jm_^d^ O_jm_), where J_jm_^d^ ∼ Bernoulli(0.4) (1 activates the shift), O_jm_ ∼ DU{−3,…,3}, and bd_y_ clamps to fiscal year y before advancing Saturday/Sunday to Monday. A_jm_ = round₂(a_j_ [1 + J_jm_^A^ ε_jm_]), where J_jm_^A^ ∼ Bernoulli(β_j_) (1 activates amount jitter) and ε_jm_ ∼ U(-r_j_,r_j_). Tax and posting lines are deterministic. Document date, posting date, and the calendar day of the entry timestamp all equal the realized expense date; entries are recorded at 09:00 plus a deterministic offset of a few minutes per employee or expense type. Case 2 therefore has no document-to-entry lag.	Case 2
T7	Accrual and deferral sequence	For c ∈ {estimate,difference,contract}, independently A_c_ = round₂(a_c_ [1+ε_c_]), ε_c_ ∼ U(−0.12,0.12). The corresponding base amounts are EUR 1380, 70, and 12,000. Tax, invoice gross, and the deferred 10/12 share are deterministic. Non-closing dates use the T6 date operator. Closing posting/document dates remain fixed; their entry target dates use the T6 date operator and are recorded at 09:00.	Case 2.1; one fiscal-year boundary
T8	Reversal and credit note	A single uniformly permuted candidate pool is partitioned without overlap: the first 30 originals form the reversal sample and the next 30 the credit-note sample. Follow-up date d_i_′ = bd(d_i_^orig^ + L_i_), L_i_ ∼ DU{5,…,25}; bd advances Saturday/Sunday to Monday. Entry time is 09:00. Line amount A_ik_′ = round₂(s_i_ A_ik_^orig^) with the debit/credit flag of each original line inverted, where s_i_=1 for reversals and s_i_ ∼ U(0.2,0.5) for credit notes; cent imbalances are added to the largest line on the short side. The original user is retained with probability p (0.90 reversal; 0.95 credit note); otherwise a user is drawn uniformly from U_f_ excluding the original. Reversal payments are removed. A credit-note payment is reduced and moved by DU{1,…,10} days without weekend correction.	Case 2.2; one fiscal-year boundary

**Table 4:** Mapping of simulation cases to the distribution rules.

Case(s)	Generated or modified object	Distribution rule	Case parameters and dependencies
1.1–1.10	Regular purchase/sales originals and their linked payments	T1-T4	ρ_g_=νg=λ_g_=0. Weekday/month weights, users, quantity rule, account structure, and n are given in Table 4. Payments use T3 and do not redraw user, item, or amount.
1.11/1.12	Purchase original and bank clearing	T1-T4	ρ_g_=νg=0.05; λ_g_=0.10 for the original. The clearing entry uses λ_g_=0 and inherits its original's attributes.
1.13/1.14	Purchase original and cash clearing	T1-T4	ρ_g_=ν_g_=0.05; λ_g_=0.15 for the original. The clearing entry uses λ_g_=0 and inherits its original's attributes.
1.15/1.16	Sales original and bank clearing	T1-T4	ρ_g_=ν_g_=0.05; λ_g_=0.10 for the original. The clearing entry uses λ_g_=0 and inherits its original's attributes.
1.17/1.18	Single-item sales original and cash clearing	T1-T4	ρ_g_=ν_g_=0.05; λ_g_=0.20 for the original. The clearing entry uses λ_g_=0 and inherits its original's attributes.
1.19	Selected year-end purchase originals and linked clearing entries	T5	C contains originals from 1.1, 1.7, 1.11, and 1.13 posted 12–01 to 12–31. n = 100; new entry window 01–01 to 01–15; clearing delay DU{1,…,7}.
1.20	Selected late sales originals and linked clearing entries	T5	C contains originals from 1.5, 1.9, 1.15, and 1.17 posted 10–01 to 12–31. n = 50; new entry window 01–15 to 03–31; clearing delay DU{1,…,10}.
1.21	Selected originals linked to high bank/cash clearing rows	T5	C contains originals posted 10–01 to 12–31 whose linked row posts at least EUR 1500 to account 1000, 1100, or 1200, excluding originals already moved by cases 1.19 and 1.20. n = 50; entry window 01–01 to 02–28; clearing delay DU{1,…,7}.
2 (payroll)	Payroll postings	T6	Deterministic payroll schedule and amounts; user Alice. The T1 candidate-timestamp pool does not apply.
2 (recurring expenses)	Recurring operating expenses	T6	Date-shift probability 0.40. Base amounts, amount-jitter probabilities, relative ranges, nominal days, tax rates, and month coverage are reported in Table 4, Simulation ID 2.
2.1	Accrual and deferral	T7	One three-entry sequence of each kind at the 2023/2024 fiscal-year boundary; user Alice. Nominal and fixed dates are reported in Table 4.
2.2	Reversal and credit note	T8	Candidate originals are purchase invoices from 1.1, 1.3, 1.11, and 1.13 posted 12–01 to 12–31. The two samples contain 30 non-overlapping originals each. U_f_ = {Alice, Bob, David, Max}.

The distributions and operators are defined as follows: DU(A): discrete uniform distribution on finite set A; U(a,b): continuous uniform distribution on (a,b); Poisson(μ): count distribution with mean μ; N(μ,σ²): normal distribution with mean μ and variance σ²; TN[a,b](μ,σ²): that normal distribution truncated to [a,b]; Bernoulli(p): binary indicator equal to 1 with probability p; SRSWOR(C,n): simple random sample of size n from C without replacement; trunc₀: truncation toward zero; floor: greatest-integer function; round₂: rounding to cents; date(x): calendar-date part of timestamp x; Pr: probability; |K|: cardinality of set K. Primitive draws are independent only where no conditioning, inheritance, shared pool, or without-replacement rule is stated. The generator uses a deterministic seed schedule derived from master seed 42. The symbolic keys are explained in Table 5.

**Table 5:** Symbol key for Tables 3, Table 4, Table 6.

Symbol	Meaning	Symbol	Meaning
i; g; d; m; y	journal entry; case group; calendar day; scheduled month; fiscal year	j (T6); c (T7); k (T8)	recurring-expense type; amount component; posting line
orig; clr; wd; mo	labels for original, linked clearing, weekday, and month	τ; τ′	candidate timestamp; dummy timestamp used only in the T1 sum
N_g,d_; B_g,d_; Z_g,d_	candidate count; weekend activation gate; weekend count draw	ρ_g_; ν_g_; H; T_g_	weekend-activation probability; non-working-hour probability; hour; realized timestamp pool
E_i_; P_i_; D_i_	entered timestamp; posting date; document date	w_wd,g_; w_mo,g_	weekday and month sampling weights for group g
X_i_; Δ_i_; λ_g_	continuous lag draw; signed whole-day lag; long-lag mixture probability	R_y_; bd_y_; bd	T2 calendar correction; T6 year-aware business-date operator; T8 weekend-to-Monday operator
K; K_i_; U_g_; U_i_	item set; selected item; group user set; selected user	Q_i_; π_i_; δ_y_; A_i_^net^	quantity; configured unit cost/price; year price multiplier; net amount
C_g_; n_g_; S_g_	candidate-original set; requested sample size; realized sample	[a_g_,b_g_]; L_i_; ℓ_g_^min^; ℓ_g_^max^	new-entry window; clearing/follow-up delay; minimum and maximum delay
d_jm_^0^; d_jm_*; J_jm_^d^; O_jm_	nominal/realized expense date; date-shift indicator; day offset	a_j_; A_jm_; J_jm_^A^	base amount; realized recurring amount; amount-jitter indicator
β_j_; ε_jm_; r_j_	amount-jitter probability; relative jitter draw; maximum relative range	a_c_; A_c_; ε_c_	base amount, realized amount, and jitter draw for T7 component c
A_ik_^orig^; A_ik_′; s_i_	original/follow-up line amount; reversal or credit share	p; U_f_	original-user retention probability; follow-up-user set

The calculation consists of the following steps: First, a pool of candidate entry timestamps is generated for each fiscal year and case group. This separates regular working hours from non-working hours. Secondly, the entered date is selected from that pool using weekday and month weights to emphasize particular days or months. Thirdly, a whole-day lag is drawn and applied backwards from the entered date to obtain the document date and the posting date, followed by calendar corrections for weekends and the fiscal year boundary. The fourth step involves shifting the *entered_date* for a subset of year-end transactions into the subsequent fiscal year, while keeping the *posting_date* in the closing period.

In Table 6, an overview of the generated postings is given. As shown, different postings (cases) are defined, with specific parameter settings based on our predefined business cases. For example, in case 1.3/1.4, purchase postings were generated where Thursdays and Fridays have a slightly higher chance to be selected. By doing this, we tried to consider different posting behavior. Users are selected randomly for each posting. Another example is shown in case 1.5/1.6 where we generated postings where only one item was sold (single), but also multiple items of the same type (multiple). In case 1.7/1.8, for example, we generated only transactions within a specific time period. Using different weights, we simulated that more postings are made in the summer months. Finally, cases 1.11 to 1.18 include a delay component that draws a larger time delta between the entry date and the document date, whereas in cases 1.1 to 1.10 this component is switched off and all postings are entered promptly. This allowed us to include postings entered with lower promptness. In sum, we generated 102,429 business transactions (256,232 posting lines),^1^ distributed almost evenly across the two fiscal years (51,064 and 51,365 transactions). All transactions consist of two, three, or four posting lines.

**Table 6:** Overview of generated postings.

Simulation ID (Jupyter Notebook)	Case Type	Description of parameters of posting	Number^a^
1.1	Purchase merchandise	Accounts: 3400, 1576, 1600. Entry window: 01–01 to 12–31; weekday weights (Mon-Sun)=(1,1,1,1,1,0,0); month weights=1. Users: Alice, Bob, David, Max. Quantity Q∼DU{1,…,20}.	5000
1.2	Payment	Accounts: 1600, 1100. One linked clearing entry per Case 1.1 original; amount, user, tax rate, and text are inherited; dates follow T3.	5000
1.3	Purchase merchandise	Accounts: 3400, 1576, 1600. Entry window: 01–01 to 12–31; weekday weights=(1,1,1,1.5,1.5,0,0); month weights=1. Users: Alice, Bob, David, Max. Quantity Q = 1.	4000
1.4	Payment	Accounts: 1600, 1000. One linked clearing entry per Case 1.3 original; amount, user, tax rate, and text are inherited; dates follow T3.	4000
1.5	Sales of goods	Accounts: 1400, 8400, 1776. Same timestamp pool as Case 1.1. Entry window: 01–01 to 12–31; weekday weights=(1,1,1,1,1,0,0); month weights=1. Users: Alice, Bob, David, Max. 2000 entries use Q = 1; 1500 use Q∼DU{1,…,5}.	3500
1.6	Incoming payment	Accounts: 1100, 1400. One linked clearing entry per Case 1.5 original; amount, user, tax rate, and text are inherited; dates follow T3.	3500
1.7	Purchase merchandise	Accounts: 3400, 1576, 1600. Candidate window: 03–31 to 10–31; weekday weights=(1,1,1,1,1,0,0). Month weights for Mar-Oct=(1.1,1.2,1.3,1.5,1.7,1.7,1.3,1.2). Users: Alice, Bob, David. Q∼DU{1,…,20}.	4000
1.8	Payment	Accounts: 1600, 1100. One linked clearing entry per Case 1.7 original; amount, user, tax rate, and text are inherited; dates follow T3.	4000
1.9	Sales of goods	Accounts: 1400, 8400, 1776. Same timestamp pool as Case 1.7. Users: Alice, Bob. Quantity Q∼DU{1,…,5}.	4000
1.10	Incoming payment	Accounts: 1100, 1400. One linked clearing entry per Case 1.9 original; amount, user, tax rate, and text are inherited; dates follow T3.	4000
1.11	Purchase merchandise	Accounts: 3400, 1576, 1600. Entry window: 01–01 to 12–31; weekday and month weights=1. Users: Bob, Charlie, Fred. Q∼DU{1,…,20}. Weekend gate ρ=0.05; non-working-hour probability ν=0.05; long-lag probability λ=0.10.	2000
1.12	Payment	Accounts: 1600, 1100. One linked clearing entry per Case 1.11 original. Attributes are inherited; the clearing dates use T3 with λ=0.	2000
1.13	Purchase merchandise	Accounts: 3400, 1576, 1600. Same timestamp and user pool as Case 1.11. Q∼DU{1,…,20}; ρ=0.05; ν=0.05; λ=0.15.	1500
1.14	Payment	Accounts: 1600, 1000. One linked clearing entry per Case 1.13 original. Attributes are inherited; the clearing dates use T3 with λ=0.	1500
1.15	Sales of goods	Accounts: 1400, 8400, 1776. Same timestamp and user pool as Case 1.11. Q∼DU{1,…,5}; ρ=0.05; ν=0.05; λ=0.10.	1000
1.16	Incoming payment	Accounts: 1100, 1400. One linked clearing entry per Case 1.15 original. Attributes are inherited; the clearing dates use T3 with λ=0.	1000
1.17	Sales of goods	Accounts: 1400, 8400, 1776. Same timestamp pool and users as Case 1.11, including weekend and non-working-hour generation. Q = 1; ρ=0.05; ν=0.05; λ=0.20.	500
1.18	Incoming payment	Accounts: 1000, 1400. One linked clearing entry per Case 1.17 original. Attributes are inherited; the clearing dates use T3 with λ=0.	500
1.19	Cross-period: year-end purchases	Originals from Cases 1.1, 1.7, 1.11, and 1.13 posted 12–01 to 12–31; linked clearings are from 1.2, 1.8, 1.12, and 1.14. New original entry window: 01–01 to 01–15 of the following year; clearing delay: DU{1,…,7} days.	100
1.20	Cross-period: late sales	Originals from Cases 1.5, 1.9, 1.15, and 1.17 posted 10–01 to 12–31; linked clearings are from 1.6, 1.10, 1.16, and 1.18. New original entry window: 01–15 to 03–31; clearing delay: DU{1,…,10} days.	50
1.21	Cross-period: high bank/cash	Originals posted 10–01 to 12–31 whose linked clearing row posts at least EUR 1500 to account 1000, 1100, or 1200, excluding originals already moved by cases 1.19 and 1.20. New original entry window: 01–01 to 02–28; clearing delay: DU{1,…,7} days.	50
2	Different expenses	Normal-generator accounts: 1000, 1100, 1576, 4100, 4120, 4130, 4210, 4240, 4250, 4260, 4360, 4580, 4600, 4710, 4930, 4950. User: Alice. Payroll is deterministic; recurring expenses follow T6^b^. Account 1200 is introduced only by the later shadow-bank anomaly.	288 (2023); 289 (2024)
2.1	Period-end adjustment: accrual	Accounts: 4240, 1700, 2020, 1576, 1600, 1100. Closing estimate: posting/document date 12–31; entry target 02–05. Actual invoice: nominal date 02–08; payment: nominal date 02–20. Entry targets/nominal dates use the T6 date operator. Base estimate EUR 1380 and difference EUR 70, each with ±12% continuous jitter; tax 19%; user Alice.	3
2.1	Period-end adjustment: deferral	Accounts: 4260, 1576, 1100, 0980. Contract: nominal date 11–15. Deferral: fixed posting/document date 12–31; entry target 01–22. Release: fixed posting/document date 01–01; entry target 01–25. Targets/nominal dates use the T6 date operator. Base contract EUR 12,000 with ±12% jitter; deferred share 10/12; user Alice.	3
2.2	Reversal	Purchase originals from Cases 1.1, 1.3, 1.11, and 1.13 posted 12–01 to 12–31. First 30 entries of one uniform random permutation; no overlap with credit notes. Full reversal; original payment removed. Follow-up delay DU{5,…,25} days and roll to Monday-Friday. Original-user retention probability: 0.90.	30
2.2	Credit note	Next 30 entries of the same random permutation. Credit share s∼U(0.2,0.5); remaining amount paid. Follow-up delay DU{5,…,25} days and roll to Monday-Friday. Original-user retention probability: 0.95. Remaining payment moved by DU{1,…,10} days without weekend correction.	30

a Unless stated otherwise, *Number* refers to journal entries generated per fiscal year. Cases 1.19–1.21, 2.1, and 2.2 are instantiated once at the 2023/2024 fiscal-year boundary. The actual number is lower because (i) offsetting entries with a posting date after the last simulated fiscal year are omitted, and (ii) Case 2.2 removes the payment for each canceled original transaction.

b Recurring expense order: rent, utilities, cleaning, maintenance, insurance, vehicle costs, advertising, packaging materials, office supplies, legal/consulting. Base amounts a_j_=(22,500; 1450; 620; 900; 1300; 780; 1200; 850; 260; 1800); nominal days=(3; 5; 7; 12; 15; 18; 20; 22; 24; 26); tax rates=(0; .19; .19; .19; 0; .19; .19; .19; .19; .19); amount-jitter probabilities β_j_=(0; .8; .5; .6; 0; .7; .7; .8; .9; .4); relative ranges r_j_=(0; .12; .10; .25; 0; .18; .25; .15; .30; .35). Insurance occurs only in January; all other types recur monthly. Office supplies are paid in cash; all others through bank. December 2023 utilities are omitted from Case 2 and represented by the accrual sequence in Case 2.1. The corresponding configuration is the recurring list in Section 2 of synthetic_data_4203.ipynb.

Next, we generated synthetic anomalies by injecting attribute combinations that do not occur in the normal journal-entry population. The injections were aligned with our four JET scenarios and the two additional anomaly patterns and applied only to postings that were originally labeled as normal (label = 0) and not already part of prior anomaly injections. Unless stated otherwise, the counts below refer to one fiscal year, and anomalies at each count were injected independently into both simulated years. For J1, we selected 60 postings from the 1000 highest-amount candidates and modified account and user attributes to create unusual combinations. For J2, we restricted candidates to cash postings with amounts greater than EUR 1500 and altered 30 postings by changing the user. For J3, we used time-delta classes of 0–9 days (promptly), 10–29 days (less promptly), and 30+ days (not at all promptly), and injected anomalies only into the less-promptly and not-at-all-promptly groups (60 postings each). For J4, we added two time-based anomaly types: non-working-hours postings in underrepresented user patterns (50 postings) and weekend postings with unusual user/account context (125 postings). For A1, we rerouted 30 postings to a rarely used second bank account, and for A2, we redirected the reference of 20 clearing postings (drawn across both fiscal years) to a business transaction of an unrelated category. All modified postings were labeled as anomalies (label = 1) and retained subtype information via marking. The marking attribute encodes the subtypes (usually in combination with an unusual user): 1 = high amount, 2 = high cash disbursement, 3 = delayed entry (10–29 days), 4 = very delayed entry (>29 days), 5 = non-working hours, 6 = weekend, 7 = shadow bank account, 8 = cross-linked clearing; marking 0 denotes normal postings. In total, this resulted in 850 anomalous journal entries out of 102,429 total (0.83%). By design, six of the eight anomaly scenarios were injected into the entry populations that a JET actually returns, since the auditor's task is to discriminate within such a selection list rather than to search the entire general ledger. This creates a circularity between injection and evaluation, but it concerns candidate selection rather than the injected signal: the rule populations are considerably larger than the sets of injected cases they contain—323 anomalies among 15,694 weekend entries, and 190 among 402 high-cash entries—so plain rule matching reaches a recall close to one at a precision of only 2.1% to 47.3% and therefore serves as a baseline, not as a detector. Bias-free evaluation consequently requires conditioning on a given audit population, in which the rule is constant and carries no discriminative information, excluding the pre-computed rule attributes promptly, weekend, nwh, top_n, and high_cash from the feature set. The remaining two scenarios, marking = 7 (shadow bank account) and marking = 8 (cross-linked clearing), were deliberately injected without conditioning on any JET: about two-thirds of them fall outside every JET result population, and none of the tests targets them (marking 7: 63.3%; marking 8: 70.0%). They therefore provide a rule-independent control group whose detection cannot be explained by rule matching.

**Fig. 1:**
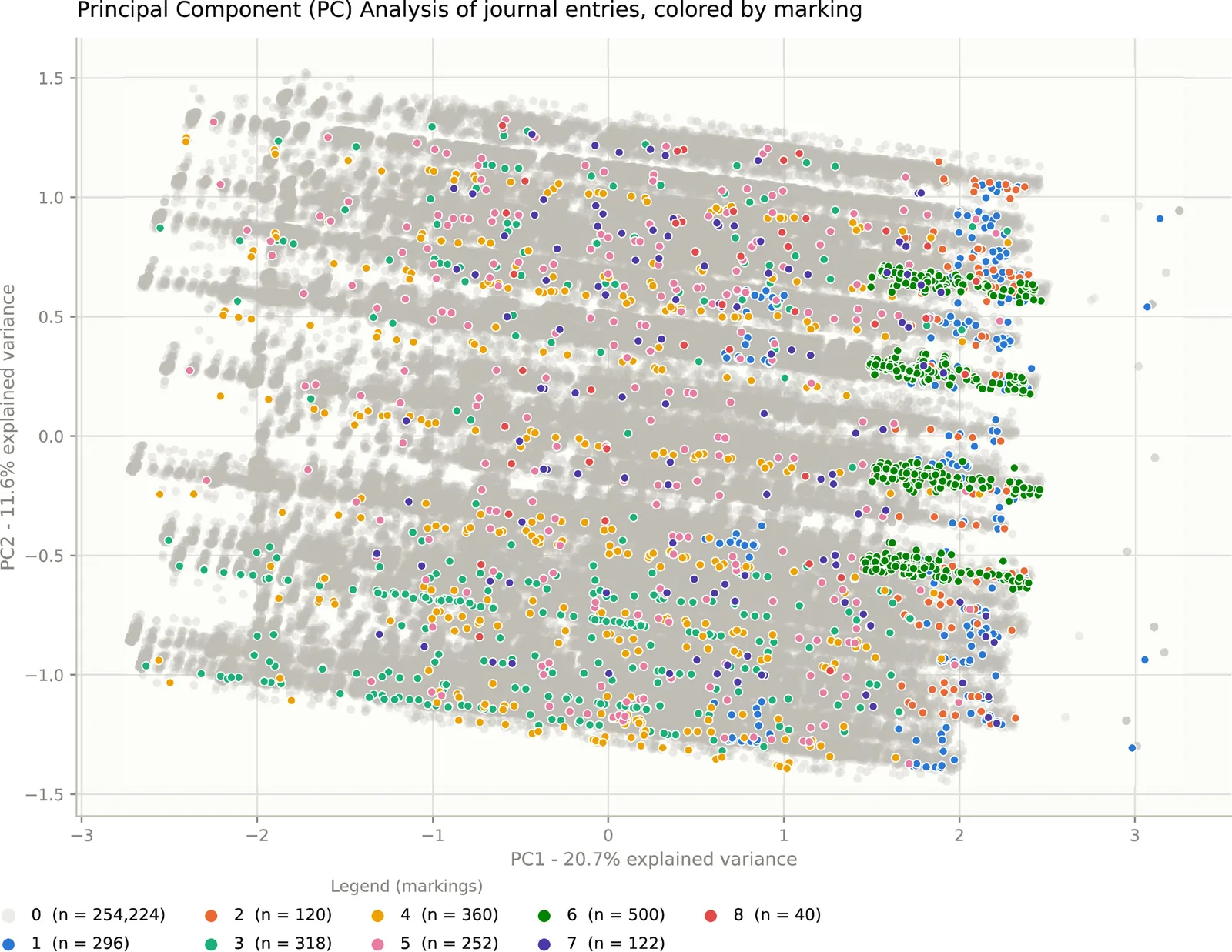
Principal Component (PC) Analysis of journal entries, colored by marking.

To verify that the injected anomalies are statistically visible, we performed a principal component analysis (PCA) as a minimal sanity check; it is not intended as a benchmark. The preprocessing is implemented in the final section of *synthetic_data_4203.ipynb* at the posting-line level: the categorical features *gl_account, cd_flag, user, tax_rate,* and *ref_id* were one-hot encoded; the numerical feature *amount* was log-transformed and standardized. Entry timestamps were transformed via cyclic encoding of hour and weekday, and the log-transformed difference between *entered_date* and *document_date* was added to capture large gaps between occurrence and entry. *gl_account_name* was excluded due to its perfect correlation with *gl_account, posting_id* as a pure identifier, and the free-text field *text*. The indicators *promptly, weekend, nwh, top_n, high_cash, label*, and *marking* were excluded to avoid label leakage. Fig. 1 shows the projection, colored by marking. The band structure reflects the large share of discrete features. As expected, some anomaly patterns separate distinctly from regular postings, while the most challenging ones would require dedicated feature engineering to become salient – mirroring real-world accounting data, in which anomalies occur at varying levels of detection difficulty.

To assess how far the reported figures depend on a particular random draw, the generation workflow was repeated with nine random seeds. Dataset size is essentially seed-independent (102,413 ± 24 journal entries, 256,201 ± 47 posting lines), and the injection budget fixes the anomaly count at 850 (0.83%) in every run. Both indicators are constant by construction and are therefore not evidence of stability. What the seed varies is which entries are affected: the JET result populations remain stable in size, as reported here for the weekend and the high-cash test, which bracket the range as the largest population at the lowest precision and the smallest at the highest (15,713 ± 116 and 420 ± 14 entries)—rule precision spans 2.07% ± 0.03 to 45.1% ± 1.7, and the lowest recall stays at 97.9% ± 0.7. The published dataset (with a seed of 42) lies within this range of each indicator, so the reported baseline is a property of the generator rather than of a single execution.

## Limitations

The dataset was generated based on simplified assumptions and reflects only a clearly defined real-world posting context. In addition, simulated postings are characterized by randomly generated values. The underlying probability distributions could have been further refined to better represent the specific business cases under consideration or to simulate different posting behaviors more realistically. Furthermore, the variability of generated values regarding accounts, users, and posting amounts could be enhanced to more accurately capture the complexity of real-world accounting processes. This would allow for the inclusion of more demanding posting scenarios, such as discounts, goods complaints, or ancillary postings, thereby adding greater depth and realism to the dataset. Moreover, it remains unclear to what extent anomalous journal entries can be reliably detected, as anomalies may follow a wide range of patterns, and transaction types often exhibit distinct behavioral characteristics. Fraudulent entries are frequently designed to remain inconspicuous or to resemble legitimate postings. Consequently, the transactional context in which postings occur must be carefully considered when interpreting the results. Finally, further transaction types could be added to broaden the transactional variety.

## Ethics Statement

The authors confirm that they have read and adhered to the ethical requirements for publication in Data in Brief. The current work does not involve human subjects, animal experiments, or data collected from social media platforms.

## CRediT author statement

**Jan Gronewald:** Conceptualization, Methodology, Software, Data curation, Writing - original draft. **Sebastian Stephan:** Conceptualization, Methodology, Software, Data curation, Writing - original draft. **Alexander Rombach:** Conceptualization, Methodology, Writing - original draft. **Peter Fettke:** Supervision, Conceptualization, Methodology, Writing - review and editing.

## Footnotes

1 Payment postings are deliberately recorded a few days after the corresponding invoice, so invoices from December of the last simulation year receive an entered_date in the following year. The filter cuts off this overhang so that the dataset covers exactly the two simulated fiscal years. The clearing postings removed in the process leave a number of open items in the last simulation year.
